# Hypereosinophilia is a predictive biomarker of immune checkpoint inhibitor-induced hypopituitarism in patients with renal cell carcinoma

**DOI:** 10.1186/s12902-022-01024-4

**Published:** 2022-04-26

**Authors:** Hodaka Yamada, Satoshi Washino, Daisuke Suzuki, Rika Saikawa, Shiori Tonezawa, Rie Hagiwara, Shunsuke Funazaki, Masashi Yoshida, Tsuzumi Konishi, Kimitoshi Saito, Tomoaki Miyagawa, Kazuo Hara

**Affiliations:** 1 Department of Endocrinology and Metabolism, Jichi Medical University Saitama Medical Center, 1-847, Amanuma-cho, Omiya-ku, Saitama, 330-8503 Japan; 2 Department of Urology, Jichi Medical University Saitama Medical Center, 1-847, Amanuma-cho, Omiya-ku, Saitama, 330-8503 Japan

**Keywords:** Immune checkpoint inhibitors, Immune-related adverse events, Hypopituitarism, Renal cell carcinoma

## Abstract

**Background:**

This study aimed to evaluate whether hypereosinophilia is a clinical biomarker of immune checkpoint inhibitor-induced hypopituitarism in patients with renal cell carcinoma treated with nivolumab plus ipilimumab.

**Methods:**

This was a retrospective cohort study conducted at Jichi Medical University Saitama Medical Center between January 2018 and December 2020. In total, 12 patients with renal cell carcinoma who presented with immune checkpoint inhibitor-induced hypopituitarism were enrolled in this study. The clinical parameters and symptoms at baseline, last visit, and onset of hypopituitarism were analyzed.

**Results:**

The median period from the initial treatment with immune checkpoint inhibitors to the onset of hypopituitarism was 82.5 (range: 56–196) days. Most patients developed hypopituitarism within 6 months. One patient presented with hypophysitis and 11 patients presented with isolated adrenocorticotropic hormone deficiency. The major symptoms noted at onset were fatigue (66.7%) and loss of appetite (41.7%). None of the patients had symptoms during the last visit. However, four developed hypereosinophilia. Eosinophil fraction (%) and eosinophil count (/µL) increased during the last visit and at the onset of hypopituitarism, respectively. The serum sodium and plasma glucose levels were similar.

**Conclusions:**

The eosinophil count increased before the onset of hypopituitarism. Thus, hypereosinophilia can be an early predictor of hypopituitarism.

**Supplementary Information:**

The online version contains supplementary material available at 10.1186/s12902-022-01024-4.

## Introduction

Immunotherapy with immune checkpoint inhibitors (ICIs), such as monoclonal antibodies (mAbs), targeting the programmed cell death protein 1 (PD-1), its ligand (PD-L1), and cytotoxic T-lymphocyte antigen 4 (CTLA-4) is the new standard treatment for cancers, such as lung cancer, malignant melanoma, gastric cancer, lymphoma, urothelial carcinoma, and renal cell carcinoma (RCC) [1–3]. ICIs can affect the activated immune system and improve the prognosis of patients with cancers. However, such treatment can cause side effects, referred to as immune-related adverse events (irAEs), in several organs [4]. The major irAEs include immune-related endocrinopathies, such as hypopituitarism, thyroiditis (hypothyroidism and hyperthyroidism), and autoimmune diabetes [5]. Hypothyroidism can likely occur in patients receiving anti-PD-1 and anti-PD-L1 therapies. Meanwhile, hypopituitarism and secondary adrenal insufficiency are more common in patients receiving anti-CTLA-4 therapy [4–6]. Anti-CTLA-4 therapy combined with anti-PD-1 therapy is the first-line treatment for advanced RCC [7]. Compared with sunitinib, a multiple tyrosine kinase inhibitor, combination therapy with ICIs is associated with a better overall survival (OS) of intermediate- and high-risk (based on the prognostic risk score) patients with advanced RCC [8]. Additionally, hypopituitarism is more common in patients receiving ICI combination therapy than in those receiving ICIs alone [2, 9]. Hypopititarismcauses secondary adrenal insufficiency, and a delayed diagnosis leads to adrenal crisis. Generally, adrenal insufficiency has specific symptoms, including general fatigue, headache, loss of appetite, weight reduction, and nausea [10–12]. Early diagnosis of ICI-induced hypopituitarism is clinically important for the initiation of appropriate treatment and prevention of adrenal crisis, which has a fatal clinical course [11, 12]. A latest survey revealed that adrenal crisis accounted for 10% of all deaths of patients with adrenal insufficiency [13]. Hydrocortisone replacement therapy can be used to treat ICI-induced hypopituitarism, and ICI therapy can be resumed once the patient’s condition stabilizes [14]. However, studies on ICI-induced hypopituitarism in patients with all types of tumors, including RCC, are limited. Predicting the development of hypopituitarism in the early stage via routine laboratory screening and based on symptoms during follow-up is clinically useful for patients with RCC receiving ICI combination therapy. Adrenal insufficiency due to hypopituitarism is known to cause hyponatremia and hypoglycemia [11]. These biochemical changes are not so common in ICI-induced hypopituitarism; conversely, hypereosinophilia was observed before the onset of hypopituitarism [15]. Several studies have reported that eosinophilia is associated with the occurrence of any grade of irAEs [16–18]. Thus, the present study aimed to evaluate whether hypereosinophilia can be used as a useful early clinical biomarker of ICI-induced hypopituitarism in patients with RCC.

## Methods

### Study design and patients

**Fig. 1:**
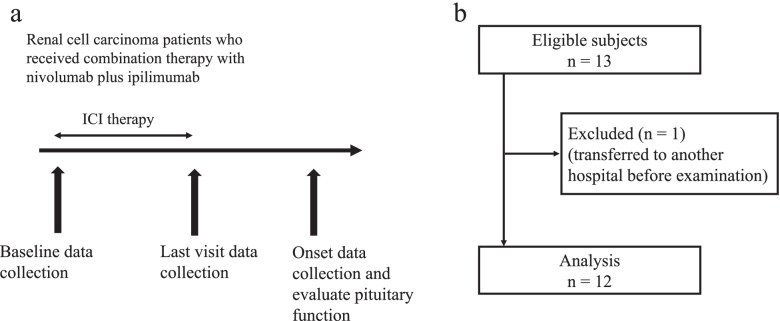
Study protocol (**A**) and flow chart of the cohort (**B**)

This retrospective cohort study was conducted at a single center. Figure 1 shows the study protocol and flow chart. We enrolled Japanese patients with RCC diagnosed with ICI-induced hypopituitarism at the Jichi Medical University Saitama Medical Center between January 2018 and December 2020. Then, data regarding clinical characteristics at the baseline (before the start of combination therapy with ICIs), last visit (before the onset of hypopituitarism), and onset of hypopituitarism were collected. The inclusion criteria were as follows: patients who received combination therapy with nivolumab, anti-PD-1 mAb, ipilimumab, and anti-CTLA-4 mAb and those with pituitary–adrenal dysfunction and ICI-induced endocrinopathy diagnosed by endocrinologists using not only the basal hormone test based on the management guidelines for ICI-induced irAEs in endocrine organs established by the Japanese Endocrine Society but also the endocrinological diagnostic test [14]. Additionally, the exclusion criteria were as follows: patients with primary and secondary adrenal insufficiency, those who received ICI therapy before, those who received corticosteroid therapy for another disease, those with two or more concomitant cancers, and those who transferred to another hospital before the definitive diagnosis of hypopituitarism. This study was approved by the ethics committee of the Jichi Medical University, Saitama Medical Center (no. S19-177) and performed according to the ethical guidelines of the Declaration of Helsinki.

### Data collection

We obtained baseline demographic data comprising age, sex, body mass index (BMI), ICI therapy duration, prior treatment history with radical nephrectomy, previous medical history, pituitary magnetic resonance imaging (MRI) findings, and symptoms at onset of hypopituitarism via patient interviews or medical records. We used the Common Terminology Criteria for Adverse Events (CTCAE, version 5.0) to classify irAE severity. Blood data, such as blood cell count; biochemical laboratory findings; and endocrinological hormone data, such as serum cortisol, adrenocorticotropic hormone (ACTH), thyroid-stimulating hormone, free triiodothyronine, and free thyroxine levels, were also collected. Undetectable serum cortisol and ACTH levels were 0.9 µg/dL and 1.5 pg/mL, respectively. Hyponatremia was defined as a serum sodium level of < 135 mmol/L, hyperkalemia as a serum potassium level of > 5.5 mmol/L, hypoglycemia as a plasma glucose level of < 70 mg/dL, and hypereosinophilia as an eosinophil count of > 500/µL. Two types of hypopituitarism are known, hypophysitis and isolated ACTH deficiency [19]. In hypophysitis, pituitary gland enlargement and dysfunction in the production of multiple pituitary hormones are observed, whereas in isolated ACTH deficiency, no pituitary gland enlargement occurs and only ACTH deficiency is observed [19].

### Statistical analysis

Data are presented as mean ± standard deviation, and skewed variables are expressed as median with interquartile ranges. The clinical characteristics of the three groups were compared using Friedman’s test to analyze for continuous variables and Cochran’s Q test. Post hoc analyses were conducted using Bonferroni’s test. Characteristics of the two groups were compared using paired or Student’s *t*-test. OS was analyzed using the Kaplan–Meier method. Survival rates and 95% confidence intervals (CIs) were calculated using log-rank test. All statistical analyses were conducted using EZR (Jichi Medical University Saitama Medical center) [20]. A *p*-value of < 0.05 was considered statistically significant.

## Results

### Characteristics of patients

Initially, 13 patients were eligible for inclusion in the study; however, 1 patient who transferred to another hospital before the diagnosis of ICI-induced hypopituitarism was excluded. Finally, 12 patients with RCC treated with nivolumab plus ipilimumab were enrolled in this study (Fig. 1b). Table 1 shows the basic characteristics of the patients. All the patients complained of different symptoms including fatigue (66.7%) and appetite loss (41.7%) upon the onset of hypopituitarism. However, none of them presented with symptoms during the last visit. One patient was diagnosed with adrenal crisis, and most patients presented with CTCAE grade 2 or 3 disease. One patient had an enlarged pituitary gland on MRI and hypophysitis, which was characterized by not only ACTH deficiency but also pituitary hormone (gonadotroph and thyrotroph) deficiency. In total, 11 patients presented with isolated ACTH deficiency but not pituitary enlargement. Another irAE observed was thyroiditis (41.7%). Four patients had antithyroid antibodies (antithyroid peroxidase antibodies and antithyroglobulin antibodies), and all of them presented with thyroiditis. Seven (58.3%) patients presented with hypopituitarism, and five (41.7%) presented with two or more irAEs (Supplemental Fig. 1a). The median period from the initial treatment to the onset of hypopituitarism was 82.5 (range: 56–196) days. Five patients presented with symptoms within 2–3 months from the initial administration of ICIs, and most patients presented with symptoms within 6 months (Supplemental Fig. 1b).

**Table 1:** Clinical characteristics of patients

Age (years)	65 [62–79]
Male sex (%)	8 (67)
BMI (kg/m^2^)	22.1 ± 4.3
Histology (clear cell carcinoma/chromophobe renal cell carcinoma), n (%)	11 (92)/1 (8)
Radical nephrectomy, n (%)	9 (75)
Course of ICI therapy, n (%)	
2	1 (8.3)
3	4 (33.3)
4	7 (58.3)
Duration (days) from the initial ICI therapy to the onset of symptoms	82.5 [67–100]
Symptoms at onset, n (%)	
Fatigue	8 (66.7)
Loss of appetite	5 (41.7)
Lightheadedness/hypotension	3 (25)
Nausea/vomiting	3 (25)
Weight loss	2 (16.7)
Weakness	2 (16.7)
Joint pain	1 (8.3)
Muscle pain	1 (8.3)
Adrenal crisis	1 (8.3)
GTCAE grade, n (%)	
1	1 (8.3)
2	5 (41.7)
3	5 (41.7)
4	1 (8.3)
Pituitary enlargement, n (%)	1 (8.3)
Type of hypopituitarism, n (%)	
Hypophysitis	1 (8.3)
Isolated ACTH deficiency	11 (91.7)
Other irAEs, n (%)	
Thyroiditis (hypothyroidism)	5 (41.7)
Hepatitis	2 (16.7)
Another pituitary hormone deficiency	1 (8.3)
Colitis	1 (8.3)
Pneumonitis	1 (8.3)
Myocarditis	1 (8.3)
Fulminant-type 1 diabetes	1 (8.3)
Positivity for antithyroid Abs, n (%)	4 (33.3)

Data were expressed as mean ± standard deviation, and skewed variables as medians with interquartile ranges

*Abs* antibodies, *BMI*, body mass index, *ICIs* immune checkpoint inhibitors (nivolumab plus ipilimumab combination therapy), *CTCAE* Common Terminology Criteria for Adverse Events, *ACTH* adrenocorticotropic hormone

**Fig. 2:**
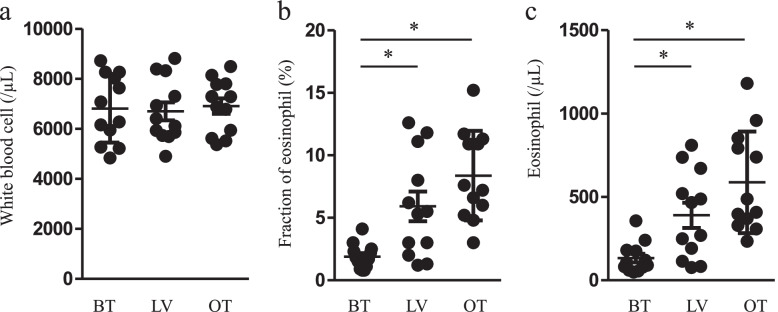
Distribution of white blood cell count (**A**), fraction of eosinophil (**B**), and eosinophil count (**C**) at baseline (BT), last visit (LV), and onset of symptoms (OT). Eosinophil fraction (%) and eosinophil count were significantly higher at LV and OT than at BT. Data were expressed as scatter plots with a bar (mean ± standard error of the mean). *p < 0.05, after post hoc analysis

**Fig. 3:**
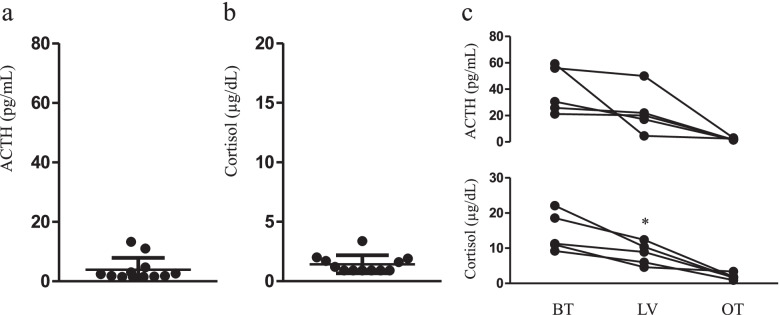
The adrenocorticotropic hormone (ACTH) (**A**) and cortisol levels (**B**) at the onset of symptoms are shown. There were changes in the ACTH and cortisol levels of five patients at baseline (BT), last visit (LV), and onset of symptoms (OT) (C). The cortisol level at LV was significantly lower than that at BT. Data were expressed as scatter plots with bar (mean ± standard error of the mean). *p < 0.05 vs. BT by paired *t*-test

Table 2 shows the laboratory data. There were no changes in white blood cell counts at the baseline, last visit, and onset of hypopituitarism. Meanwhile, eosinophil fraction (%) and eosinophil count (/µL) significantly increased during the last visit and at the onset of hypopituitarism, respectively (Fig. 2). The serum sodium, serum potassium, and plasma glucose levels, which are often altered in hypopituitarism, did not differ. Five patients had hypereosinophilia (> 500/µL) at onset but none had it at baseline (Table 2). Figure 3a and b show the serum cortisol and plasma ACTH (samples collected in the morning) levels at onset. The serum cortisol (< 0.9 µg/dL) and plasma ACTH levels (< 1.5 pg/mL) of six and three patients, respectively, were undetectable. The serum cortisol and plasma ACTH levels of five patients (annual blood and hormone tests) were followed up. The serum cortisol levels during the last visit decreased (*p* = 0.022). However, the plasma ACTH level did not change (*p* = 0.187) (Fig. 3c). The survival rate at 35 months was 90.9% (95% CI, 50.8%–98.7%) (Supplemental Fig. 2).

**Table 2:** Transition of clinical parameters in patients with ICI-induced hypopituitarism

	Baseline	Last visit	Onset	*p*-value
WBC count (/µL)	6805 ± 1359	6698 ± 1253	6905 ± 1076	0.919
Neutrophil count (/µL)	4903 ± 1121	4295 ± 1291	4022 ± 874	0.153
Lymphocyte count (/µL)	1294 ± 474	1560 ± 371	1738 ± 531	0.076
Eosinophil count (/µL)	99 [74–177]	389 ± 261	588 ± 304	< 0.001
Sodium level (mmol/L)	139 ± 2.1	139 ± 3.1	137.5 [135–138]	0.056
Potassium level (mmol/L)	4.58 ± 0.33	4.42 ± 0.42	4.29 ± 0.28	0.148
Glucose level (mg/dL)	118 ± 27	124 ± 35	95 [82–106]	0.059
Blood urea nitrogen level (mg/dL)	17.2 ± 3.5	20.5 ± 3.5	18.9 ± 6.9	0.265
Creatinine level (mg/dL)	0.93 [0.86–1.00]	1.15 ± 0.26	1.15 [1.07–1.32]	0.184
Hyponatremia, n	0	2	3	0.247
Hyperkalemia	0	0	0	1.000
Hypoglycemia, n	0	0	2	0.135
Hypereosinophilia, n	0	4	5	0.015

Data were expressed as means ± standard deviation, and skewed variables as medians with interquartile ranges

*WBC* white blood cell

## Discussion

Hypopituitarism is an endocrinopathy caused by ICIs, particularly anti-CTLA-4 mAb. The incidence rate of anti-CTLA-4 mAb-induced hypopituitarism is approximately 6%–8% as per previous reports [2, 5, 9]. Moreover, the incidence rate of anti-PD-1/PD-L1-induced hypopituitarism is lower than that of anti-CTLA-4 mAb-induced hypopituitarism [5]. Combination therapy with nivolumab and ipilimumab, rather than ipilimumab monotherapy, has been associated with a higher prevalence (7.7%–10%) of hypopituitarism [9, 21]. Recently, Kobayashi et al. revealed that the incidence rate of ipilimumab-induced hypopituitarism was 24% and that of nivolumab and pembrolizumab (anti-PD-1 mAb)-induced hypopituitarism was 6% in patients with malignant melanoma or non-small cell lung carcinoma (NSCLC) [19]. Therefore, hypopituitarism is a common endocrinopathy [19]. In relation to this finding, we must pay attention to ICI-induced hypopituitarism, particularly that occurring at the start of combination therapy with ICIs. We observed the development of hypereosinophilia without symptoms at the last visit (before the onset of ICI-induced hypopituitarism). During routine hormone assessments, five patients had a lower serum cortisol level during the last visit than at baseline (Fig. 3c). It is still unknown whether ICI-induced hypopituitarism can develop immediately or gradually. In Ariyasu et al.’s study, five patients presented with ICI-induced hypopituitarism. Moreover, three patients had an elevated eosinophil count before the onset of hypopituitarism [15]. Taken together, the development of hypereosinophilia before the onset of symptoms, which is caused by ICI-induced hypopituitarism, might reflect a subacute decrease in plasma cortisol level. Furthermore, there were no differences between patients with and those without hypereosinophilia in terms of clinical parameters (Supplemental Table). Hyponatremia and hypoglycemia are signs of adrenal insufficiency [11]. In this study, the incidence of hypereosinophilia was higher than that of hyponatremia and hypoglycemia. Thus, in asymptomatic cases, hypereosinophilia might be a useful predictor of hypopituitarism, and its identification might be beneficial to prevent a life-threatening adrenal crisis.

Other than ICI-related endocrinopathy, the most common complication was hypothyroidism (Table 1). In this study, one patient was diagnosed with adrenal crisis. This patient presented with thyroiditis and received levothyroxine (LT-4) before the development of hypopituitarism. In patients with concomitant adrenal insufficiency and hypothyroidism, glucocorticoid replacement should be started to treat acute adrenal insufficiency and crisis [22]. The blockade of both CTLA-4 and PD-1 was associated with a high risk of irAEs including hypopituitarism and thyroiditis [2, 23]. Therefore, when we treat patients with hypothyroidism using LT-4, progress should be cautiously monitored if there is no sign of adrenal insufficiency.

In our study, 11 (> 90%) of 12 patients developed hypopituitarism within 5 months after treatment. Several reports have revealed that the duration from treatment to ICI-induced hypopituitarism was shorter in patients treated with anti-CTLA-4 mAb therapy or combination therapy with ICIs than in those who received anti-PD-1 mAb therapy [9, 10]. Moreover, a recent study showed that the onset duration of hypophysitis (mean: 56 days) was shorter than that of isolated ACTH deficiency (mean: 162 days) in patients with hypopituitarism [19]. Only one patient with hypophysitis was included in our study, and the onset duration was 68 days after the initial administration of ICIs. Patients with hypophysitis presented with pituitary gland enlargement and two or more pituitary anterior lobe hormone deficiency [19]. Hence, when hypopituitarism is suspected, MRI and tests other than pituitary anterior lobe hormone screening should be conducted.

ICI-induced hypopituitarism is correlated with longer OS of patients with NSCLC and those with malignant melanoma [19]. In patients with NSCLC, ICI-induced thyroiditis was found to be associated with the positive efficacy of nivolumab [24, 25]; however, the association between ICI-induced hypopituitarism and prognosis in patients with RCC is still unknown. Ikeda et al. reported that in patients with RCC who received nivolumab plus ipilimumab as the first-line therapy, the occurrence of irAEs was significantly associated with progression-free survival but not OS [26]. However, we could not evaluate these oncological clinical outcomes and compare nonpituitary irAEs (Supplemental Fig. 2). It is possible that the presence of nonpituitary irAEs and the number of irAEs may have influenced the prognosis; hence, future studies are needed for further clarification.

The American Society of Clinical Oncology Clinical Practice Guideline recommended ACTH and cortisol work-up for patients in whom adrenal insufficiency is suspected [27]. Based on our study, we propose that if clinical symptoms are absent, ACTH and cortisol levels should be checked in patients with elevated eosinophils. In our study, ICI-induced hypopituitarism occurred within 6 months after the initiation of ICI combination therapy in almost all patients. For patients receiving ICI combination therapy, monitoring ACTH and cortisol levels for at least 6 months might also facilitate the early diagnosis of hypopituitarism. Preliminary data suggested that eosinophilia was a predictor of the future onset of irAEs [16]. We could not evaluate the association between eosinophilia and the onset of other irAEs. It is possible that other prior irAEs, such as thyroiditis, affected eosinophil counts [16].

The present study had several limitations. First, it included a small number of participants. Moreover, it was retrospective in nature and conducted at a single center. Second, a random blood test was performed daily in the morning. However, the cortisol level was not obtained during fasting. Blood tests were performed at different situations for patients. Third, long-term follow-up of the endocrine system and prognosis could not be performed. Additionally, a follow-up pituitary MRI was not conducted after treatment. All patients were treated with hydrocortisone at physiological doses. Thus, more large-scale studies conducting long-term follow-ups must be performed in the future.

In conclusion, hypereosinophilia was observed before the development of symptoms in patients with RCC treated with combination therapy with ICIs. Thus, this condition might be a useful predictive biomarker of ICI-induced hypopituitarism and could be used in the early diagnosis of secondary adrenal insufficiency.

## Supplementary Information

**Additional file 1.** Clinical characteristics between patients with ICI-induced hypopituitarism who presented with hypereosinophilia and those who did not

**Additional file 2:** **Supplemental Figure 1.** Total number of irAEs perpatient (A) and duration (days) from the initial administration of nivolumabplus ipilimumab to the onset of symptoms (B).

**Additional file 3:** **Supplemental Figure 2. **Overall survival (OS) after startingcombination therapy with nivolumab plus ipilimumab in patients with renal cellcarcinoma and immune checkpoint inhibitor-induced hypopituitarism. OS at 35 months was 90.9% (95% CI, 50.8%–98.7%).

## Data Availability

The data generated during the current study are not publicly available due to ethical restrictions, but are available from the corresponding author on reasonable request.
