# Supplementary material for: Hypereosinophilia is a predictive biomarker of immune checkpoint inhibitor-induced hypopituitarism in patients with renal cell carcinoma
Source: BMC Endocr Disord. 2022 Apr 26;22:110. doi: 10.1186/s12902-022-01024-4 (PMC9040214; doi:10.1186/s12902-022-01024-4)
Supplement: Supplementary file 1 — Additional file 1. Clinical characteristics between patients with ICI-induced hypopituitarism who presented with hypereosinophilia and those who did not [file 12902_2022_1024_MOESM1_ESM.docx]

Additional file 1. Clinical characteristics between patients with ICI-induced hypopituitarism who presented with hypereosinophilia and those who did not

|  | Patients with hypereosinophilia (n = 5) | Those without (n = 7) | p-value |
| --- | --- | --- | --- |
| Age (years) | 62 [61–64] | 72.1 ± 7.9 | 0.142 |
| Male sex (%) | 5, (100) | 3 (43) | 0.081 |
| BMI (kg/m^2^) | 22.1 ± 3.73 | 22.2 ± 5.00 | 0.969 |
| Duration (days) from the initial ICI therapy (days) to the onset of symptoms | 88 [68–95] | 86.4 ± 31.4 | 0.684 |
| WBC count (/µL) | 7522 ± 644 | 6465 ± 1142 | 0.094 |
| Neutrophil count (/µL) | 4098 ± 715 | 3969 ± 1025 | 0.814 |
| Lymphocyte count (/µL) | 1862 ± 712 | 1649 ± 397 | 0.522 |
| Eosinophil count (/µL) | 904 ± 174 | 362 ± 81 | < 0.001 |
| Sodium level (mmol/L) | 137.6 ± 2.51 | 134.3 ± 5.94 | 0.272 |
| Potassium level (mmol/L) | 4.26 ± 0.25 | 4.31 ± 0.31 | 0.756 |
| Glucose level (mg/dL) | 86 ± 22 | 97 [86.5–111] | 0.432 |
| Blood urea nitrogen level (mg/dL) | 18.8 ± 5.54 | 19.8 ± 8.22 | 0.963 |
| Creatinine level (mg/dL) | 1.14 [1.13–1.16] | 1.22 ± 0.40 | 0.876 |
| ACTH level (pg/mL) | 1.8 [1.8–3.0] | 2.4 [1.5–3.65] | 0.623 |
| Cortisol level (ug/dL) | 0.9 [0.9–1.2] | 1.41 ± 0.50 | 0.665 |

Data were expressed as means ± standard deviation (SD), and skewed variables as medians with interquartile ranges. WBC, white blood cell; ACTH, adrenocorticotropic hormone.
