# Supplementary material for: Hypereosinophilia is a predictive biomarker of immune checkpoint inhibitor-induced hypopituitarism in patients with renal cell carcinoma
Source: BMC Endocr Disord. 2022 Apr 26;22:110. doi: 10.1186/s12902-022-01024-4 (PMC9040214; doi:10.1186/s12902-022-01024-4)
Supplement: Supplementary file 2 — Additional file 2: Supplemental Figure 1. Total number of irAEs perpatient (A) and duration (days) from the initial administration of nivolumabplus ipilimumab to the onset of symptoms (B). [file 12902_2022_1024_MOESM2_ESM.ppt]

## Slide 1
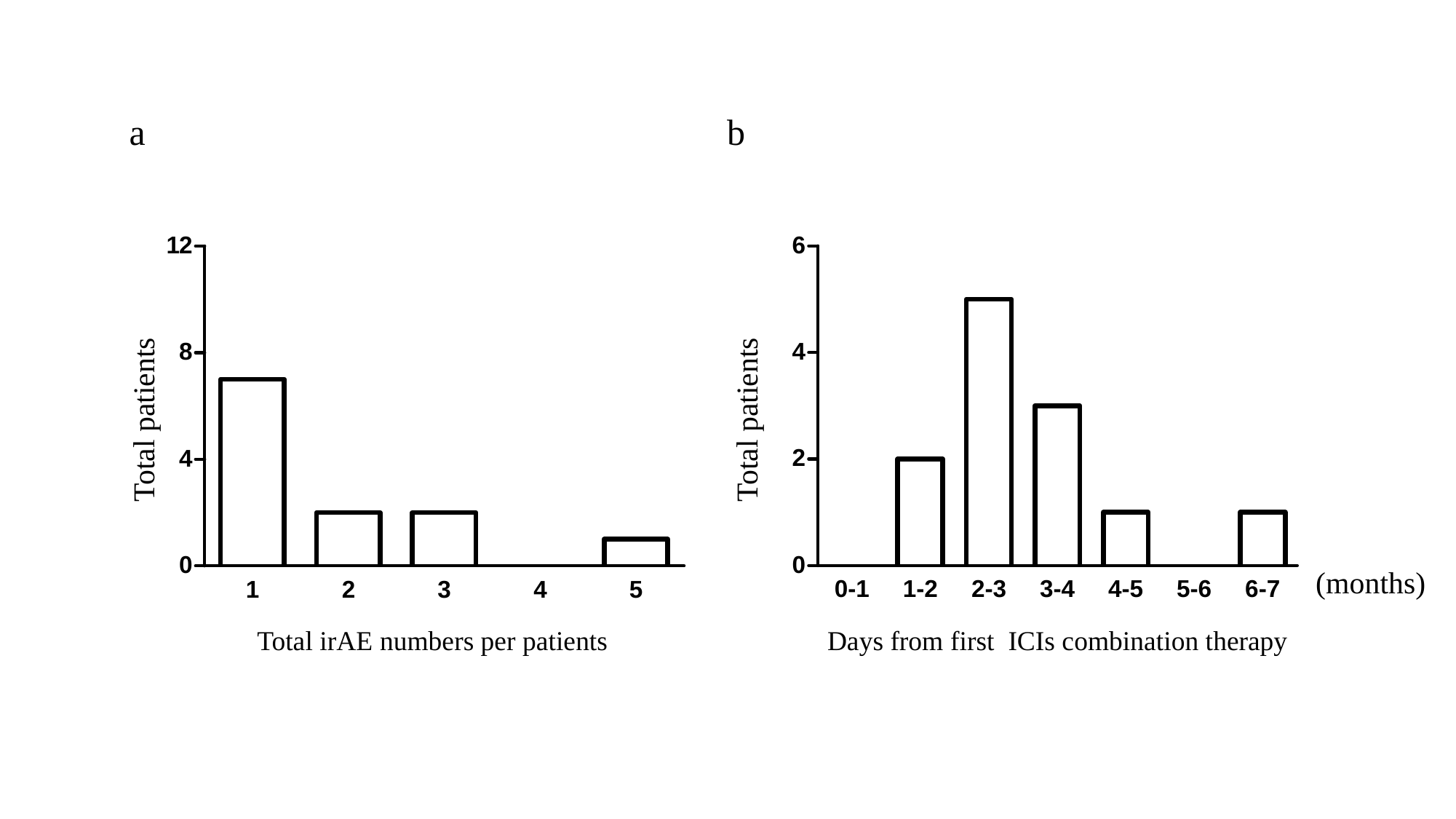

a
b
Total patients
Total patients
(months)
Total irAE numbers per patients
Days from first ICIs combination therapy
