# Supplementary figures and images for: Hypereosinophilia is a predictive biomarker of immune checkpoint inhibitor-induced hypopituitarism in patients with renal cell carcinoma
Source: BMC Endocr Disord. 2022 Apr 26;22:110. doi: 10.1186/s12902-022-01024-4 (PMC9040214; doi:10.1186/s12902-022-01024-4)

## Slide 1
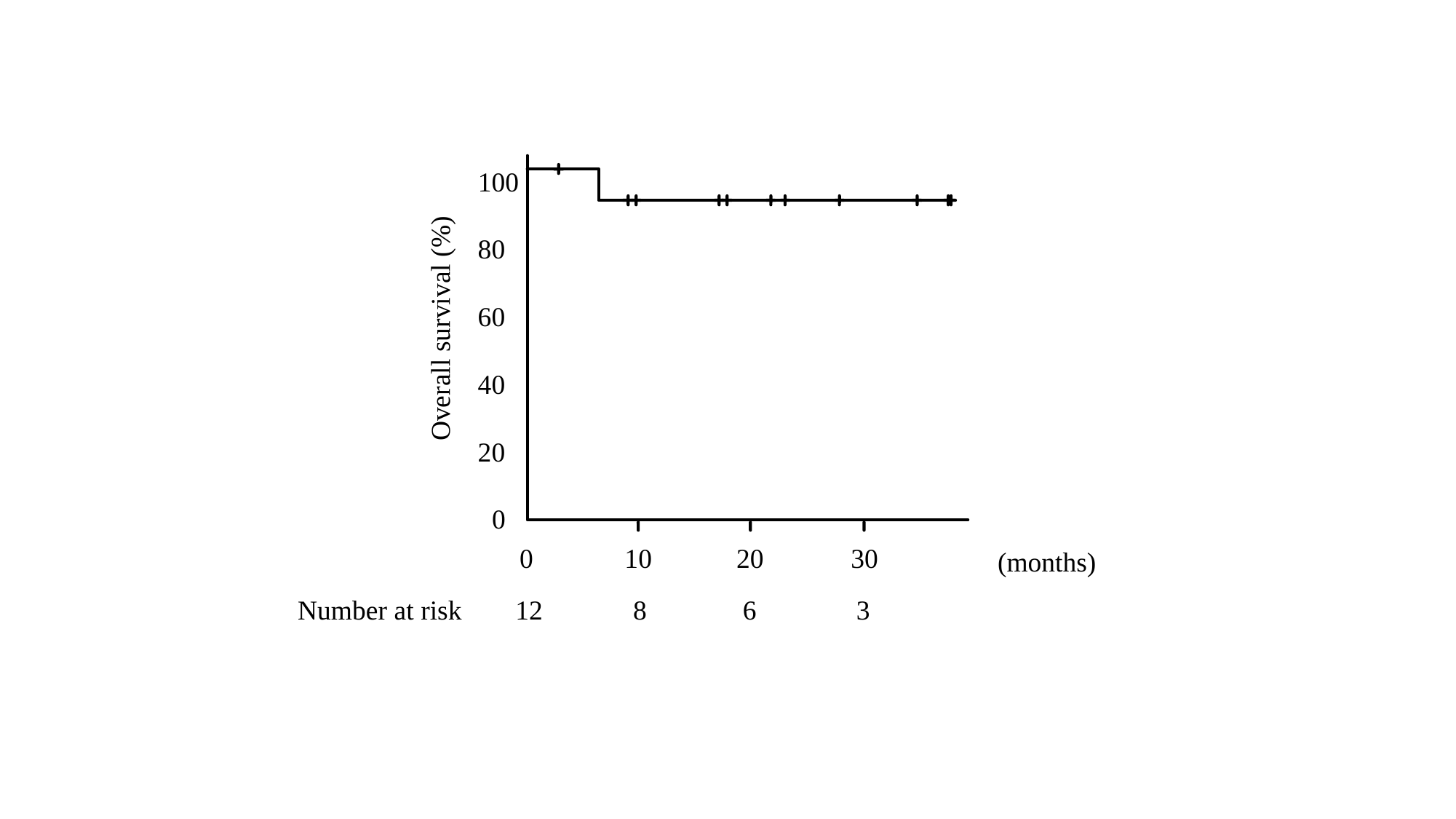

100
80
60
40
20
0
　Overall survival (%)
0
10
20
30
(months)
Number at risk
12
8
6
3

Supplement: Supplementary file 3 — Additional file 3: Supplemental Figure 2. Overall survival (OS) after startingcombination therapy with nivolumab plus ipilimumab in patients with renal cellcarcinoma and immune checkpoint inhibitor-induced hypopituitarism. OS at 35 months was 90.9% (95% CI, 50.8%–98.7%). [file 12902_2022_1024_MOESM3_ESM.ppt]
